# THE EFFECTIVENESS OF NON-PHARMACOLOGICAL INTERVENTIONS ON SLEEP QUALITY IN RA AND FMS: A SYSTEMATIC REVIEW

**DOI:** 10.1093/geroni/igad104.3637

**Published:** 2023-12-21

**Authors:** Ching-I Chin

**Affiliations:** New York University, New York City, New York, United States

## Abstract

**Objective:**

Sleep disturbances can significantly impact the well-being of individuals with Rheumatoid Arthritis (RA) and Fibromyalgia (FMS), leading to daytime sleepiness, fatigue, and increased pain. This systematic review aims to assess the available evidence on the effectiveness of non-pharmacological interventions in improving sleep quality for individuals with RA and FMS.

**Method:**

A comprehensive search was conducted in English electronic databases (CINAHL plus, SPORTDiscus, EMBASE, Medline, PubMed, Web of Science, and Google Scholar) to identify relevant articles. The review includes 15 studies, consisting of 13 randomized controlled trials (RCTs) and 2 non-randomized controlled trials (non-RCTs), published between 2012 and 2022. Primary and secondary outcome measures related to sleep quality were assessed using subjective and objective measures.

**Results:**

Interventions in the studies were diverse, involving physical and mind-body approaches, lasting 6 to 16 weeks with session durations of at least 30 minutes. The total intervention hours ranged from 6 to 32 hours across all studies. Follow-up evaluations were conducted in four studies up to 6 months after the last treatment. While the Arthritis Foundation Exercise Program and Yoga did not report statistically significant improvements in sleep quality for RA, non-pharmacological interventions showed a positive impact on sleep outcomes for RA and FMS populations.

**Conclusion:**

Non-pharmacological interventions show promise as a potential alternative method to improve sleep quality for individuals with RA and FMS. However, there is no evidence to support the superiority of one intervention over another. Keywords: Rheumatoid Arthritis. Fibromyalgia Syndrome. Sleep quality. Sleep disturbance. Exercise. Non-pharmacological intervention.

